# Distinct Antifouling Mechanisms on Different Chain Densities of Zwitterionic Polymers

**DOI:** 10.3390/molecules27217394

**Published:** 2022-10-31

**Authors:** Clil Regev, Zhongyi Jiang, Roni Kasher, Yifat Miller

**Affiliations:** 1 Department of Chemistry, Ben-Gurion University of the Negev, P.O. Box 653, Be’er Sheva 84105, Israel; 2 Department of Desalination and Water Treatment, Zuckerberg Institute for Water Research, Jacob Blaustein Institutes for Desert Research, Ben-Gurion University of the Negev, Midreshet Ben-Gurion 8499000, Israel; 3 Key Laboratory for Green Chemical Technology of Ministry of Education, School of Chemical Engineering and Technology, Tianjin University, Tianjin 300072, China; 4 Ilse Katz Institute for Nanoscale Science and Technology, Ben-Gurion University of the Negev, Beér-Sheva 84105, Israel

**Keywords:** grafted polymer density, antifouling, molecular simulations, poly zwitterion, hydration layer

## Abstract

Antifouling polymer coating surfaces are used in widespread industries applications. Zwitterionic polymers have been identified as promising materials in developing polymer coating surfaces. Importantly, the density of the polymer chains is crucial for acquiring superior antifouling performance. This study introduces two different zwitterionic polymer density surfaces by applying molecular modeling tools. To assess the antifouling performance, we mimic static adsorption test, by placing the foulant model bovine serum albumin (BSA) on the surfaces. Our findings show that not only the density of the polymer chain affect antifouling performance, but also the initial orientation of the BSA on the surface. Moreover, at a high-density surface, the foulant either detaches from the surface or anchor on the surface. At low-density surface, the foulant does not detach from the surface, but either penetrates or anchors on the surface. The anchoring and the penetrating mechanisms are elucidated by the electrostatic interactions between the foulant and the surface. While the positively charged ammonium groups of the polymer play major role in the interactions with the negatively charged amino acids of the BSA, in the penetrating mechanism the ammonium groups play minor role in the interactions with the contact with the foulant. The sulfonate groups of the polymer pull the foulant in the penetrating mechanism. Our work supports the design of a high-density polymer chain surface coating to prevent fouling phenomenon. Our study provides for the first-time insights into the molecular mechanism by probing the interactions between BSA and the zwitterion surface, while testing high- and low-densities polymer chains.

## 1. Introduction

Fouling is a reversibly or irreversibly phenomenon by which materials from environments adsorb non-selectively to surface materials. This phenomenon causes obstacles and problems in various fields, such as in medical applications (e.g., spread of infectious diseases, implant rejection, and malfunction of biosensors), in maritime industry, power plants, water treatment systems, and food industry [1,2,3]. The latter fields suffer from the effects of fouling on pipelines, oil rigs, heat exchangers, maricultural cages, hulls of ships and seawater intakes. Furthermore, the fouling phenomenon accelerates corrosion that eventually damages the protective surface. The materials that adsorb on the surface materials include various foulants, such as microorganisms (e.g., bacteria, fungi, and protozoans), suspended particles, organic molecules, and other macromolecules. Hence, there is a worldwide interest in designing better coating for surfaces to minimize the fouling phenomena.

Polymers are considered as one of the most likely materials for the prevention of fouling, because some possess antimicrobial properties, some can reject a wide range of foulants, and others exhibit excellent biocompatibility properties. Thus, protective coating by polymers plays a significant role in producing antifouling surfaces [4]. To date, there is a broad range of antifouling strategies that are used in the different industries. One of these strategies includes the antifouling polymer coating that consists of silicone and fluorinated polymer chemistry-based coatings and are known as non-stick antifouling coatings [5]. However, these antifouling polymer coating surfaces were difficult to commercialize, due to the limited supply, high cost, short-term efficacy, and specificity of natural antifouling compounds. Furthermore, these polymer coating surface have problems in meeting the environmental legislation requirements [6,7]. Hence, the focus shifted towards a production of polymer-based antifouling coating that are cheap, nontoxic, biocompatible, easy to process, have a wide-range efficacy, and are highly versatile. These antifouling coating are mainly based on poly(ethyleneglycol) and other hydrophilic polymers (e.g., polyacrylates and polysaccharides), but the interest in these materials to date has been shifted toward zwitterionic polymers [8,9].

The fouling resisting zwitterionic surfaces are hydrophilic, hydrogen bond forming via coordination with water molecules, and electrically neutral [10,11]. Moreover, the antifouling ability of the zwitterionic surfaces is related to the relatively high hydration and surface energy, due to the tightly bound water shell that creates a physical and free energy barrier, hence preventing adsorption of foulants [12]. The developments of zwitterionic antifouling coating surfaces are promising, because of the simplicity of their synthesis, their superior antifouling capability, their abundancy of raw materials and ease of functionalization [12]. One of the zwitterionic antifouling coating surfaces is poly-sulfobetaine methacrylate (SBMA), which is also named as poly-3-[2-(*N*-methacryloylethyl)-*N*,*N*-dimethylammonio] propane sulfonate (poly(SPE)), which is a biocompatible, [13,14,15] cheap [16,17,18,19] and easy to process [20].

The density of the zwitterionic polymer coating surfaces affects the antifouling performance. In general, it was proposed by an experimental study that high-density zwitterionic polymer coating surfaces improve the antifouling performance [21,22]. However, at specific high densities the antifouling performance is decreased [23,24]. Therefore, it is crucial to optimize and control the density of the polymer chains on the surface for producing excellent antifouling performance. One of the challenges of producing high-density zwitterionic polymer coating surfaces with excellent performance is to control the spacing between the polymer chains during the polymerization processes. Hence, there is a motivation to investigate the effect of the density zwitterionic polymer coating surfaces on the antifouling performance. To address this, computational molecular modeling tools are capable of controlling and illustrating the spacing between the polymer chains. Recently, the density of zwitterionic polymer coating surfaces has been investigated by molecular dynamics (MD) simulations using lysozyme [25] and alginate molecule [26] as foulants.

In the present study, the zwitterionic poly(SPE) polymer brush models were designed to produce high-density and low-density surfaces, using computational molecular modeling tools. The BSA protein was applied as a model foulant to assess the antifouling coating surfaces performance. BSA is abundant in nature in an extensive number of organisms [27] and has become a common foulant model in many antifouling-related studies due to its inexpensive production cost [1,28,29]. The molecular modeling tools in the current study, mimic a static adsorption experiment, by which the protein is initially positioned on the top of the surfaces. MD simulations enable us to follow the antifouling performance along the surfaces. Our work illustrates a first study that provides insights into the molecular mechanisms at the atomic resolution and accomplishing knowledge on the molecular interactions between the foulant BSA and the poly(SPE) surfaces at distinct densities. 

## 2. Computational Methods

### 2.1. Constructions of Low-Density and High-Density Polymer Chain Surfaces

**Figure 1:**
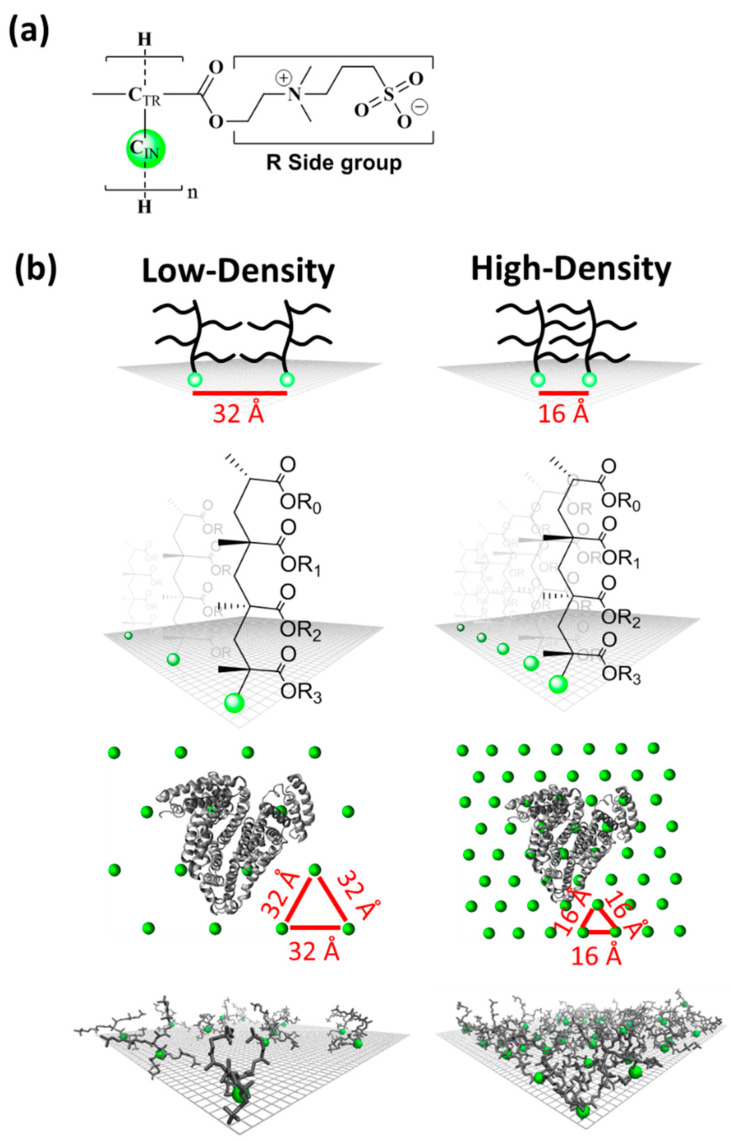
(**a**) Chemical structure of poly(SPE) repeating unit. (**b**) Top to bottom: Two types of distances that allow poly(SPE) chains to interact with each other via the R side groups, Poly(SPE) representations of one “row” and the positions for all the polymer chains within the models (bottom) of low- and high-densities surfaces. The green spheres are C_IN_ atoms of R_3_ side groups.

The polymer chain surfaces were designed by the Accelrys Discovery Studio software (BIOVIA, San Diego, CA, USA). The polymer chain surfaces are composed on monomers of 3-[2-(*N*-methacryloylethyl)-*N*,*N*-dimethylammonio] propane sulfonate (SPE). Each single poly(SPE) chain was designed with four repeated units of polymerized SPE monomers. The syndiotactic form is a common form that is produced under radical polymerization conditions of methacrylate [30,31,32,33]. Thus, the chiral carbons within the backbone of the polymer chain side groups R_0_–R_3_ were organized in a syndiotactic form, i.e., in alternating manner (Figure 1).

The size of the two-dimensional surface was designed due to the size of the chosen foulant: BSA protein. The maximal diameter size of BSA is ~70 Å. In addition, each surface was extended by 15 Å along the edges of the horizontal plane xy axis of each surface. Therefore, the dimension for each constructed surface in plane xy axis was set to ~100 × 100 Å [2]. 

To construct by computational modeling tools high- and low-density poly(SPE) coatings, it is important to allow the poly(SPE) chains to interact with each other. Previous studies modelled self-assembled monolayers of SPE and poly(SPE) on gold template with highly packed brushes [25,34]. In the current study, we examined the optimal distances in each density that will allow the poly(SPE) chains to interact with each other while maintaining minimal clashes and cluttering (Figure 1b). Hence, two distinct poly(SPE) surfaces were constructed: low-density surface and high-density surface. Each poly(SPE) surface was constructed by accommodating polymer chains with a particular distance within the two-dimensional surface (Figure 1). The distance was calculated from the C_IN_ atom of the side group R_3_ of each chain. The low-density surface consists of 16 parallel polymer chains and the distance between each one of the 16 parallel chains was set to 32 Å. The high-density surface consists of 56 parallel polymer chains and the distance between each one of the 56 parallel chains was set to 16 Å.

The system models are inspired by real synthetic procedures of grafting the polymer chains onto an existing surface template to integrate the antifouling coating homogeneously on the surface. Therefore, the polymer chains bind to specific locations across a wide template and thus the binding site lacks the freedom of mobility compared to the whole chain. By following grafting procedures and fixing the binding sites, one can prevent clustering and preserve the different densities.

### 2.2. Constructions of BSA Protein on Poly(SPE) Low-Density and High-Density Polymer Chain Surfaces

One of the foulants that is applied to investigate fouling/antifouling performance of surfaces is the BSA protein. The advantage of applying the BSA protein as a foulant model is because it contains both positive and negative charged residues on its surface domain. The charged residues allow for the investigation of the effect of charged foulants (e.g., bacteria) on both low- and high-density surfaces. Still, the total charge of the BSA demonstrates a negatively charged protein. The structure of the BSA protein that was applied in the current work is based on X-ray crystallography (PDB id code: 3V03) [35]. 

The BSA was arranged on top central location of each surface with a maximal face-to surface contacts in three different orientations: A, B and C at each one of the two density surfaces. The detailed description of each orientation is described in Table S1 and Figure S1 (Supplementary Materials). The charged residues on BSA are homogeneously dispersed on its surface domain, therefore the three orientations exhibit almost similar surface charges. For each orientation (A, B, and C), the ratios of the quantities of these types are relatively similar, therefore the surface charge for the three orientations expected to be similar. The positive:negative ratios for A, B, and C are 1.0:1.6, 1.0:1.9, and 1.0:2.0, respectively. Hence, a total of three models were constructed at a high-density surface: models HA, HB and HC, and a total of three models were constructed at low-density surface: models LA, LB and LC (Figure S2). All of these six models were constructed by the Accelrys Discovery Studio software (BIOVIA, San Diego, CA, USA).

### 2.3. Integration of the Poly(SPE) Chains within CHARMM Force-Field

The integration of the poly(SPE) chains was established by defining the parameters for the new polymer based on well-established chemical analogs that were provided by CHARMM36 force-field (mackerell.umaryland.edu (accessed on 12 September 2022)). According to its corresponding analog, each atom within a polymerized SPE unit was assigned by charge, bond length, angles, torsion and VDW. The four analogues that were used to parametrize the poly(SPE) unit were neopentane, ethyl ethanoate, tetramethylammonium and propansulfonate. The assignments of the values were made by the CGenFF generator (cgenff.umaryland.edu) and a deviation of less than 15% for all atoms within the polymer chain was obtained.

### 2.4. All-Atom Explicit Molecular Dynamic (MD) Simulations Protocol

Molecular dynamics (MD) simulations were performed for the solvated constructed models: free poly(SPE) high-density surface, free poly(SPE) low-density surface, three models in which BSA protein orientated on at high density (models HA, HB and HC), and three models in which BSA protein orientated on at low-density (models LA, LB and LC). For each one of the three BSA-poly(SPE) system models three trajectories/replicates were ran. The MD simulations were performed in NPT ensemble (i.e., in constant number of molecules, constant pressure and constant temperature) using NAMD22 software [36] with the CHARMM36 force field [37]. 

All models were energy minimized and explicitly solvated in a TIP3P water box [38,39] with a minimal distance of 15 Å from each edge of the vertical axis of the box. Solvation on the horizontal plane axis was set within the range of the poly(SPE) chains. This allows the periodic boundary conditions to mimic homogenous distributed water-surface poly(SPE) chains. All water molecules within 2.5 Å of the polymer chains and the protein were removed. Counter-ions were added at random locations to neutralize the charge within the system. The Langevin piston method [38,40,41] was applied with a decay period of 100 fs and a damping time of 50 fs was used to maintain a constant pressure of 1 atm. The temperature was controlled by a Langevin thermostat with a damping coefficient of 10 ps [38]. Short range van der Waals (VDW) interactions were calculated using a switching function, with a twin range cutoff of 10.0 and 12.0 Å. Long range electrostatic interactions were calculated using the particle mesh Ewald method with a cutoff of 12.0 Å [42,43]. For each model the PMEGridSize(X/Y/Z) was applied according to the dimensions of each model. Each PMEGridSize was set as the smallest integer that can be (1) factorized by 2, 3 or 5, and (2) PMEGridSize(X/Y/Z) > cellBasisVector(1/2/3). The equations of motion were integrated using the leapfrog integrator with a step of 1 fs. The solvated systems were energy minimized for 2000 conjugated gradient steps. The counter-ions and water molecules were allowed to move. During minimization and MD simulations, the carbon atoms of the buried methylene groups in each polymer chain were fixed (and not allowed to move) by setting the temperature factor to these atoms to zero. The minimized solvated models were heated from 250 K to 300 K for 300 ps, and then equilibrated at 300 K for 300 ps. All models ran at 300 K for 120 ns. The timescale of 120 ns was chosen, due to the convergence of all models (Figure S3). We applied MD simulations for several orientations of the protein that initially adsorbed on the surface. The different initial orientations in the MD trajectories allow us to follow the different mechanisms. Moreover, the distinct initial orientations are alternative manner to a further replica. For each surface, three orientations/replicas were applied. The choice to run MD simulations of 120 ns was because the system was converged. The analyses of the trajectories were performed at the equilibrium states of the last 100 ns of the simulations. 

### 2.5. Root-Mean Square Deviation (RMSD) Analyses

The RMSD calculations allow us to follow the structural changes that occur during MD simulations. In the RMSD calculations the distances between the position of groups of atoms at a certain frame to their reference position of initial reference frame are computed (neglecting translation and rotation) by superimposing the different frames. The RMSD calculations examine the deviation between the position of an atom on specific frame and its position at the beginning of the simulation [44]. 

The RMSD analyses along the MD simulations were calculated for one triplicate of each one of the six models: HA, HB, HC, LA, LB and LC. The RMSD analyses were computed for three distinct components for each model: the BSA protein, the poly(SPE) chains, and for the combined BSA protein-poly(SPE) chains (Figure S3). These six models demonstrated convergences of the BSA protein and the poly(SPE) chains along the MD simulations after 20 ns of the simulations, therefore all analyses that were performed in this study were computed only for the last 100 ns of the simulations.

### 2.6. Characterization of Topology Heights of the Polymer Chains on the Surfaces 

The characterization of the topography heights of the polymer chains has been calculated for the first snapshot from the MD simulations by which the system was converged (i.e., for the first 100 converged snapshots of the simulations) and the last 1 ns of the simulations (i.e., for the last 100 snapshots of the simulations). Three positions of C atoms along each polymer chain were measured along z axis for each snapshot: C_TR_ in residue R_0_, C_IN_ in residue R_1_, and C_TR_ in residue R_3_ (Figure 1). Then, two heights were computed for each chain:

Height (1) defines the difference between the position of C_TR_ in residue R_0_ and the position of the C_TR_ in residue R_3_ for each polymer chain in each snapshot:

$$\mathrm{Height}\;\left(1\right)={\left(C_{\mathrm{TR}}\right)}_{\left(R_{0}\right)}-{\left(C_{\mathrm{TR}}\right)}_{\left(R_{3}\right)}$$

Height (2) defines the difference between the position of C_IN_ in residue R_1_ and the position of the C_TR_ in residue R_3_ for each polymer chain in each snapshot:

$$\mathrm{Height}\;\left(2\right)={\left(C_{\mathrm{IN}}\right)}_{\left(R_{1}\right)}-{\left(C_{\mathrm{TR}}\right)}_{\left(R_{3}\right)}$$

In cases where the computed values Height (1) and/or Height (2) are negative, these heights get the value zero. In cases where these values are positive, the largest value was chosen to represent the height of the polymer chain. Finally, for each chain, the average height value was computed from all 100 snapshots. In microscale, it is expected that chains will present different values and homogeneousness is not expected. Even though, to confirm convergence or equilibration of the chains, one need to perform RMSD analyses for the chains. The results show that for all six models, the RMSD values for the poly(SPE) chains, were converged after 20 ns. Thus, the last 100 ns were used to calculate the averaged topography. In such cases, some of the chains adapted bended conformations, while other adapted more stretched conformations.

### 2.7. Characterization of the Root Mean Square Fluctuation (RMSF) of the Polymer Chains on the Surfaces

The fluctuations of the residues consider the deviation between the position of an atom in a specific frame and its ensemble average position along the time. Hence, the root-square-mean-fluctuation (RMSF) calculations quantifies the change in atoms compared to a reference position along the time of the MD simulations [44]. High RMSF values indicate a high flexibility of the structure. Root-mean-square-fluctuation (RMSF) analyses were calculated for the high- and low-density poly(SPE) surfaces, and for each one of the six models, in which the BSA is presence on the poly(SPE) surfaces: HA, HB, HC, LA, LB and LC. The RMSF was calculated for each polymer chain, and considered all the side groups of the poly(SPE) chains. 

### 2.8. Characterization of the Water Hydration of the Polymer Chains on the Surfaces

The water hydration was determined for each polymer chain, and for each side group along the polymer chain for the high- and low-density poly(SPE) surfaces, and for each one of the six models, in which the BSA is presence on the poly(SPE) surfaces: HA, HB, HC, LA, LB and LC. The water hydration was calculated using the ‘coor anal’ command [45]. The hydrogen atoms of the TIP3P water molecules within the cut-off distance of 3 Å from the oxygen atoms in the poly(SPE) esteric and sulfonate groups were considered in the water hydration analyses. In the simulations that were applied in the current study, using NAMD package, the simulation parameters do not affect the solvation of R_0_ group or any group.

### 2.9. Production of Surface Plots Images for Characterizations of Heights, RMSF and Water Hydration of the Polymer Chains on the Surfaces

**Scheme 1:**
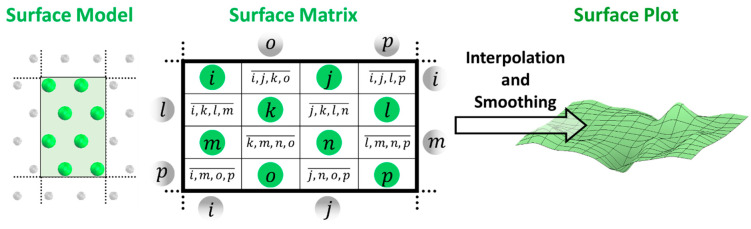
The green balls illustrate the original positions of the polymer chains on the surface. Each computed value for each chain is positioned in a numerical surface matrix. The value that was set in each cavity is the averaged value of the four surrounding polymer chains (while considering the periodic boundary conditions). Then, the matrix values are computed using the interpolation and smoothing functions.

The height, RMSF and water hydration values were calculated for each polymer chain in the surfaces. To produce a surface plot image for the characterizations of these analyses, the following procedure was applied: the position of each chain was identified in the original surface model and the value for each chain was inserted into a numerical matrix in a specific cell on the surface matrix (Scheme 1). Each cavity within the surface matrix is surrounded by four polymer chains. The value that was set in each cavity is the averaged value of the four surrounding polymer chains. For example: the value of the cavity that is surrounded by the four polymer chains *k, m, n, o* is $\bar{k,m,n,o}$.

Since in the MD simulations the periodic-boundary-conditions (PBC) are counted, the values of the PBC are considered. For example: the cavity within the surface matrix is surrounded by three polymer chains *i, k, m*. The PBC comprise the polymer chain I, therefore, the value of this cavity is $\bar{i,k,l,m}$.

Finally, the matrix values were computed with interpolation every 5 pixels and smoothed 5 times using Matlab ‘interp2′ and ‘smooth’ functions (R2020a, MathWorks, Inc.) [46,47]. The dimensions of the final surface plots in absence of the BSA for the low-density surface is ~110 × 100 Å, and for the high-density surface it is ~90 × 80 Å. The dimensions of the final surface plots in which the BSA is presence at the low-density surfaces is ~110 × 130 Å, and at the high-density surfaces it is ~120 × 110 Å.

The surface plots images were produced for three cases: (i) for average snapshots of the last 100 ns, (ii) for the first snapshot from the MD simulations by which the system was converged, and (iii) for the final snapshot from the MD simulations.

### 2.10. Estimation of Detachment and/or Adsorption of BSA on the Polymer Chain Surfaces

To estimate the detachment or the adsorption of the BSA on the polymer chains surfaces, the heights of the BSA compared to the polymer chain were computed. The positions of the C_α_ atoms of all of the residues were measured for all snapshots from MD simulations. Then, the height of the BSA was computed as the difference between the lowest C_α_ height identified in each snapshot to the lowest C_α_ height on the first snapshot:

$$\mathrm{Height}=C_{\alpha \left(\mathrm{snapshot}\right)}-C_{\alpha \left(\mathrm{initial}\right)}$$

This difference in height was chosen to characterize the detachment and/or adsorption of BSA on the polymer chain surfaces.

### 2.11. Electrostatic Interactions Analyses

The electrostatic interactions were calculated using the ‘coor contact’ command [45]. The electrostatic interactions were defined by a distance between two oppositely charged atoms, where the cut-off distance was set to 4 Å. The electrostatic interactions were computed for two types of interactions. The first type of electrostatic interaction is between charged residues within BSA (oxygen atoms of Glu and Asp, and nitrogen atoms of Arg and Lys) and the charged atoms within each polymer chain (nitrogen and three sulfonate oxygens). The second type of electrostatic interaction is the inter-chain interactions between the charged atoms within the polymer chains. 

The percentages of the occurrence of the electrostatic interactions were computed for the last 100 ns of the MD simulations, i.e., the last 10,000 snapshots of the simulations. The percentage occurrence value was obtained by dividing the total value by the number of the polymer chains in each snapshot. Then, the averaged value of all snapshots was computed. Electrostatic interactions between the BSA and the polymer chains that illustrate percentages occurrences that are less than 5% were neglected.

### 2.12. Hydrogen Bond Interactions Analyses

In our current study, we used the CHARMM’s integrated HBON function to evaluate the occurrence of H-bonding between the foulant and the surface. The HBON function considers the distance between a H-donor atom and a H-acceptor atom. The hydrogen bond interactions were calculated using the ‘coor hbon’ command [45]. The BSA polar hydrogen atoms are the donors that were considered to compute the hydrogen bond interactions. The carbonyl and the three sulfonate oxygens in the poly(SPE) chains were considered as hydrogen acceptors. The hydrogen bond interaction was defined as a distance between donor and acceptor atoms with a cut-off distance of 2.4 Å. This is the official default cutoff for hydrogen-bonding analyses that is set in the CHARMM package [45]. The percentage occurrence of hydrogen bond interactions along the MD simulations were computed.

## 3. Results and Discussion

Coating by grafted zwitterionic polymers is commonly used to improve antifouling performance of surfaces. Herein, a computational model of hydrophilic zwitterionic grafted surface is designed to investigate at the molecular level the adsorption/detachment mechanisms of the foulant BSA on surfaces with high- and low-density polymer chains. The molecular modeling and the construction of the polymer chain surfaces are detailed in the Computational Methods section and in the Supporting Information. 

### 3.1. Characterization of Surfaces with High- and Low-Density Polymer Chain Coating 

Extensive experimental studies apply antifouling coating by graft polymerization [48,49,50,51,52,53,54,55,56,57,58,59,60,61,62,63]. The complication in these experiments is that one cannot precisely determine the density of the polymer chains. During the graft polymerization process, it is difficult to produce homogeneous coating, because the number of the polymer chains and their distribution along the surface cannot be controlled. Thus, it is difficult to clarify accurately the density of the grafted polymer chains.

**Figure 2:**
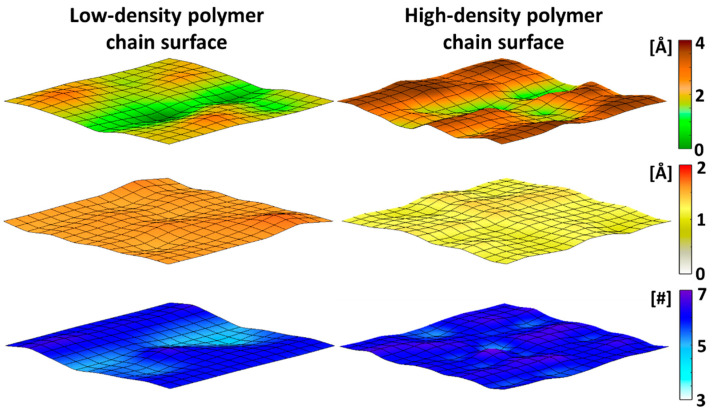
The averaged topology heights of the polymer chains (top), the RMSF of the polymer chains (middle), and water hydration of the polymer chains (bottom), i.e., number of water molecules around the polymer chains, at low-density and high-density surfaces in the absence of the foulant.

Herein, computational modeling tools were applied to design two distinct poly(SPE) surfaces: high- and low-density surfaces. The descriptions of the construction of these two surfaces are detailed in the computational methods section (Figure 1). To characterize the topology of these surfaces, the height values of each polymer chain brush were computed for each one of these two density surfaces (Figure 2). The height values of the polymer chain brushes are relatively larger for the high-density surface compared to the low-more compact density surface. This finding is predictable, because it is expected that at high-density surface the polymer chain brushes are more compact than at low-density surface. The denser surface demonstrates relatively less fluctuations, due to steric interference of nearby polymer chain brushes (Figure 2) and because of the inter-chain electrostatic interactions (Figure S4). At the low-density surface, the polymer chain brushes more spread along the surface, thus fluctuate more than at a high-density surface. Finally, the hydration shell for both high- and low-density surfaces represent similar scenario, indicating that water molecules arranged almost equivalently around the polymer chain brushes at these two densities (Figure 2).

### 3.2. BSA Does Not Penetrate at High-Density Polymer Chains but Penetrates at Low-Density Polymer Chains

The zwitterionic poly(SPE) polymer chains are characterized by hydrophilic properties, thus it is established that this zwitterionic polymer improves the antifouling performance [64,65,66]. The antifouling performance with zwitterionic polymers was extensively examined by BSA filtration and adsorption tests [21,67,68,69]. Yet, to date, the effect of the density of this zwitterionic poly(SPE) polymer chains on the antifouling performance has not been investigated. This is a first study that explores the effect of the density of the zwitterionic poly(SPE) polymer chains on the antifouling performance by BSA at the molecular level. 

**Figure 3:**
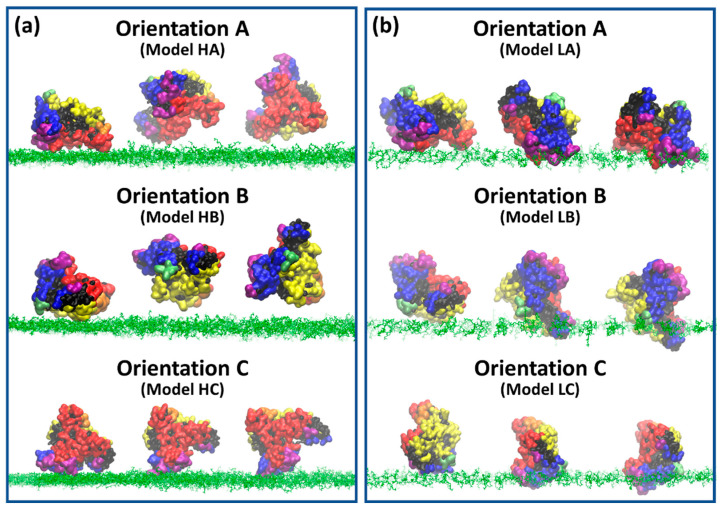
Snapshots from the MD simulations for BSA-polymer chain surfaces at 0 ns (left), 60 ns (middle), and 120 ns (right) at high-density surface (**a**) and at low-density surface (**b**) in three initial orientations designated A, B and C. Models at high-density surface are named HA, HB, and HC. Models at low-density surface are named LA, LB, and LC.

The BSA has more negatively charged residues than positively charged residues distributed on the surface (Table S1). The BSA protein that was applied was taken from a crystal structure (PDB id code: 3V03) [35]. A total of three optional orientations of the BSA were recognized to attach to the surface of the zwitterionic poly(SPE) polymer chains (Figures S1 and S2). The description of the construction of the BSA at the high- and low-density surfaces for the three orientations is detailed in the computational procedure section. Figure 3 illustrates the initial and the simulated BSA-surface models at high density: HA, HB, and HC, and at low density: LA, LB, and LC. The root mean square deviations (RMSDs) analyses of these six simulated models exhibited a convergence of the separated components: BSA and the zwitterionic poly(SPE) polymer chains after 20 ns of the simulations (Figure S3). 

**Figure 4:**
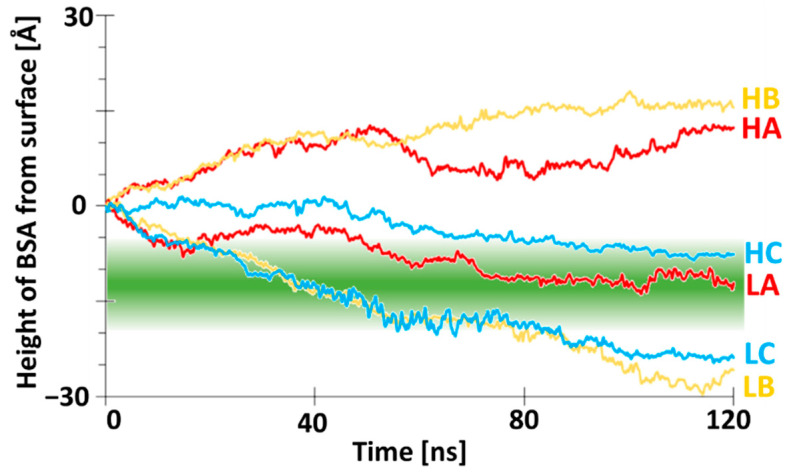
The height of the BSA from the surfaces for each model (see Figure 3 for details about the different models) along the MD simulations. The height distance values of the BSA from the surface were measured along the Z-component axis for the Cα atoms of amino acids. The values presented in the graph are taken from the lowest Cα atom of the protein. The green band represent the averaged positions of the polymer chains.

It was well-established by antifouling performance of zwitterionic polymers by BSA filtration tests that the zwitterionic polymers improve the repulsion of the BSA from the surface [21,67,68,69]. The BSA in principle binds less to the zwitterionic polymer surface. Yet, the antifouling performance has not been investigated at distinct densities of the zwitterionic polymer chains. Our simulations demonstrate that at a high density, the BSA repels from the surface at two orientations and in one orientation it only attaches the surface but does not penetrate the surface. In cases where BSA lay down in orientations A or B at high-density surface, the protein escapes along the MD simulations, while in orientation C the protein only touches the top of the surface (Figure 4 and Figure S5). At low density, the BSA adheres to the surface: in orientation A, the BSA anchors on the top of the surface, and in orientations B and C, the BSA penetrates the surface (Figure 4 and Figure S6). Thus, the antifouling performance of the poly(SPE) not only depends on the properties of the polymer chains, but also on the density of the polymer chains, and partially on the orientation of the protein.

### 3.3. Two Distinct Mechanisms of BSA at High-Density Polymer Chain Coating Surface

**Figure 5:**
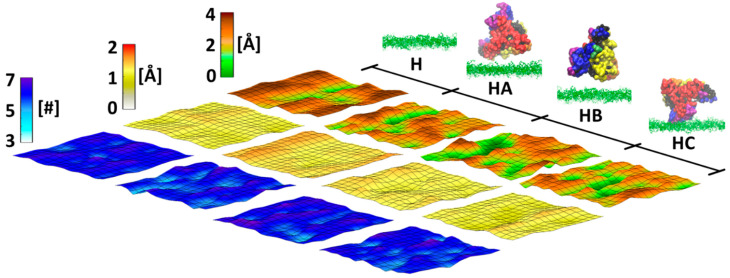
The averaged topology heights of the polymer chains (top), the RMSF of the polymer chains (middle), and the water hydration of the polymer chains (bottom) i.e., number of water molecules around the polymer chains, at high-density surface in the absence of the foulant (model H, left), and in the presence of the BSA foulant at three orientations: models HA, HB, and HC (see Figure 2 for details about the different models). The final simulated snapshots are illustrated for each model.

It is accepted that the static protein adsorption is the first event in many bio-related responses, such as biofilm formation and cell adhesion [70]. Thus, the static protein adsorption experiment is considered as an important method to explore the antifouling properties of the surface. The antifouling performance of the high-density polymer chain coating surface in the current study mimics static protein adsorption experiments. The computational modeling tools allow us to qualitatively investigate the mechanisms by which BSA adsorbs on the high-density polymer chain coating surface at different orientations. The heights of the poly chains when the BSA initially adsorbs on the surface are lower than in absence of BSA (Figure 5).

The first mechanism occurs in cases where the protein initially adsorbs at a high-density surface in orientations A and B. In these cases, the protein is detached from the surface and increases the fluctuations of the polymer chains that were not attached the protein (Figure 5). The fluctuations between the polymer chains are more pronounced than between the polymer sidechains (Figures S7 and S8). The protein initiates ‘cross-talk’ phenomena between the polymer chains that do not attach initially with the protein. These phenomena are probably due to the reorganization of the polymer brushes after the detachment of the protein. The fluctuations of the polymer brushes that do not attach initially with the protein at the end of the simulations in orientation A are higher than in orientation B. This can be explained by the proximity of the protein to the polymer brushes. Indeed, in orientation A the protein is in higher proximity than in orientation B (Figure 4). Finally, the surfaces are less hydrated than water molecules compared to the surface in absence of the protein (Figure 5), probably due to the screening of the protein that is detached from the surface.

The second mechanism appears in the case where the protein is initially attached to the surface in orientation C (Figure 5). In this orientation, the protein anchors to the surface by sticking to the top of the surface (Figure 4). The anchoring of the protein yields to less fluctuation of the surface compared to the cases the protein is detached from the surface. Moreover, the fluctuation distribution between the polymer chains in this orientation is slightly lower in the case the protein is anchored to the surface compared to the cases the protein is detached from the surface (Figure S7). The anchoring mechanisms can be clarified by contact interactions between the protein and the surface (Figure S9). The contacts between the protein and the surface include electrostatic interactions and hydrogen bond interactions. 

Finally, the surface domain by which the protein is anchored demonstrates less hydration, but in the other domains along the surface there are more hydration of water molecules (Figure 5). It is expected that the anchored protein repels the water molecules from the anchoring domain to the other parts of the surface and thus increase the hydration in those domains. The hydrations in the surfaces where the protein is detached from the surface are more inhomogeneous within the surface. It is expected that the detached protein does not affect the hydration layer. Still, more water molecules solvated the exposed R_0_ group of the polymer chains both in the cases that the protein is detached from the surface and in the case that the protein is anchored to the surface (Figure S10). 

### 3.4. Distinct Adhesion Mechanisms of BSA at Low-Density Polymer Chain Coating Surface

**Figure 6:**
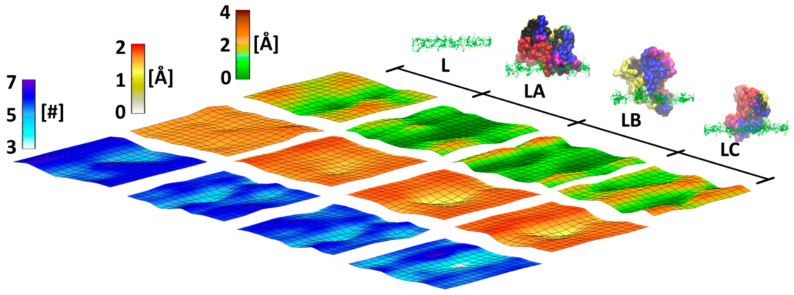
The averaged topology heights of the polymer chains (top), the RMSF of the polymer chains (middle), and the water hydration of the polymer chains (bottom) i.e., number of water molecules around the polymer chains, at low-density surface in the absence of a foulant (model L, left), and in the presence of the BSA foulant at three orientations: models LA, LB, and LC (see Figure 2 for details about the different models). The final simulated snapshots are illustrated for each model.

The adsorption of the protein was also examined at low-density polymer chain by mimicking static protein adsorption experiments. The protein was initially adsorbed at the surface in three orientations A, B, and C. While at high density, the protein was detached in orientations A and B and in orientation C the protein anchored to the surface; at low density, the protein was anchored to the surface in orientation A and was penetrated to the surface in orientations B and C (Figure 6).

The heights of the polymer chains at low density were decreased, due to the protein adsorption to the surface (Figure 6). In presence of protein, the polymer chains at the low-density surface fluctuate more than they do at high-density surfaces (Figures S7 and S8). In the cases that the protein penetrated the surface, the fluctuations in the position of the penetrating are dramatically decreased (Figure 6). The protein does not allow the polymer chains to fluctuate in the penetrating site. On the contrary, the domains around the penetrating site demonstrate relatively high fluctuations of the polymer chains. Moreover, the distribution of the fluctuations of the polymer chains when the protein is penetrating the surface is relatively higher than in the case the protein is anchored to the surface (Figure S8).

**Figure 7:**
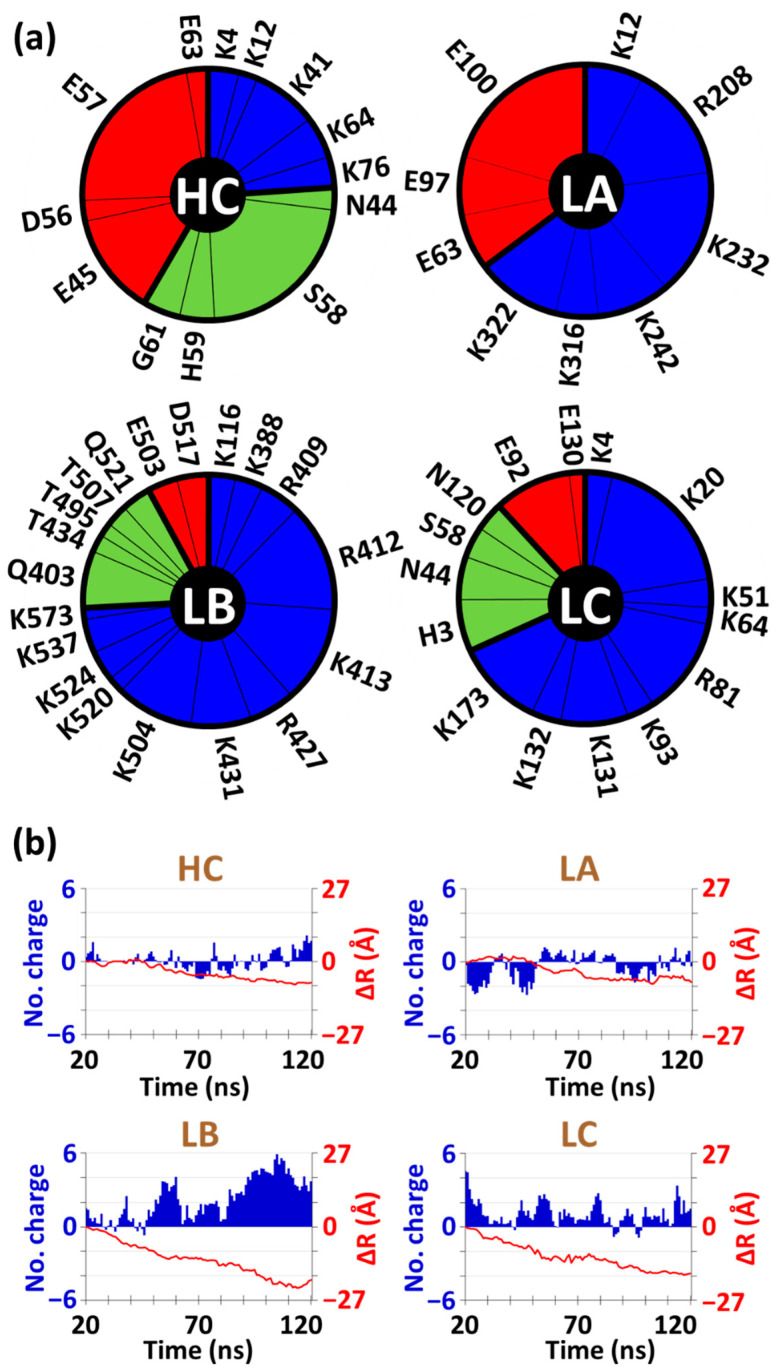
The BSA-polymer chain interactions for models HC, LA, LB, and LC, (see Figure 2 for details about the different models) by which the BSA anchors or penetrates to the surface. (**a**) The percentages of electrostatic interactions between positively charged amino acids (color: blue), negatively charged amino acids (color: red) within the BSA and the polymer chains. The percentage hydrogen bond interactions between uncharged amino acids (color: green) within the BSA and the polymer chains. The percentages were computed from the percentage values that are presented in Figure S9. (**b**) The number of positively and negatively charged amino acids (blue graphs), and the distance difference between the amino acids and the polymer chains (red graphs) along the MD simulations (20–120 ns). The large number of positively charged amino acids in models LB and LC indicate that the BSA penetrates the low-density surface (two bottom graphs).

In principle, the adsorption of proteins on polymer chain surfaces is driven by various forces, among them hydrophobic interactions, hydrogen bond interactions and electrostatic interactions. The types of the interactions between the protein and the polymer chain surface depend on the properties of both the protein and the surface. Herein, the surface consists of zwitterionic polymer chains, and the protein contains both negative and positive charged residues on the surface. In the penetrating events at low density (models LB and LC), it is more probable that the polymer chains will fluctuate more and thus more sulfonate groups are exposed. The essential interactions between the protein and the polymer chains that driven the adsorption of the protein to the inner surface domain are between the sulfonate groups of the polymer and the positively charged residues of the protein (Figure 7 and Figure S11). On the contrary, in the anchoring events at low density (model LB) and at high density (model HC), the electrostatic interactions between negatively charged residues of the protein and the ammonium groups of the polymer chain assist in docking onto the surface (Figure 7 and Figure S11). 

Finally, the hydration of the surfaces at low density in the case of the anchoring of the protein is similar to the hydration of the surface in the absence of the protein (Figure 6). In cases where the protein penetrates the surface, the hydration is decreased only at the position of the penetrating domain (Figure 6). Apparently, the penetrated protein ‘pushes’ the water molecules, thus the hydrations at the laterals are relatively more pronounced compared to the penetrating domain in the surface. 

## 4. Conclusions

Fouling is an undesirable process by which materials from the environmental, such as microorganisms, suspended particles or organic macromolecules, adhere reversibly or irreversibly to a surface. To prevent this process, one of the antifouling strategies that is commonly used is polymer coating. Zwitterionic polymers are promising candidates, because of their good chemical stability, excellent antifouling activity and that they are low-cost compounds. The grafting density of the surface, the thickness, and the flexibility of the zwitterionic polymer brushes are essential parameters that are crucial to consider when designing excellent antifouling coatings [71]. In the current study, we investigated the effect of the density of the zwitterionic poly(SPE) surface on the antifouling/fouling performance toward BSA.

Our study demonstrated that fouling/antifouling process depends on two crucial issues: (i) the density of the poly(SPE) surface, and (ii) the orientation of the initial static adsorption of the BSA. We made three characterizations to investigate the effect of the density and the orientation of the BSA on the antifouling performance: (i) the heights of the polymer chains, (ii) the RMSF of the polymer chains, and (iii) the water hydration of the polymer chains on the surfaces. These characterizations were computed in the absence of BSA and in presence of BSA at two distinct density surfaces for average snapshots (Figure 2, Figure 5 and Figure 6) and for two separated snapshots from the MD simulations: at the first snapshot of the convergence and at the final snapshot (Figures S12–S14). Interestingly, the averaged snapshots demonstrated similar results to the final snapshots. 

Our study led to four conclusions. First, while static adsorption at high-density polymer chain in two BSA orientations the BSA is detached from the surface, at low-density polymer chain the BSA is not detached from the surface in all various orientations. Second, the high-density polymer chain illustrates two distinct mechanisms that depends on the orientation of the BSA on the surface in the static adsorption test: detachment and anchoring. Third, two adhesion mechanisms were recognized at low-density polymer chain: anchoring and penetrating. Fourth, the anchoring and the penetrating mechanisms may be distinguished by the types of the electrostatic interactions between the BSA and the polymer chain surface. In cases where the BSA anchors at the top of the surface, the negatively charged amino acids within the BSA interact with the ammonium groups of the polymer chains. In cases where the BSA penetrates the surface, the positively charged amino acids within the BSA interact with the sulfonate groups on the polymer chains. The negatively charged sulfonate groups play role in dragging the BSA into the surface. The penetrating events are more likely to occur at low-density polymer chains. Hence, it is proposed to design a high-density polymer chain surface coating to prevent contaminations on the surface. 

Our work is the first study that illustrates at the atomic resolution the molecular mechanisms by which a biological contaminant, e.g., BSA protein, initially adsorbs on distinct coating density surface. Moreover, it provides insights into the molecular interactions that play role in the anchoring and the penetrating events of the BSA on the coating surfaces. Our work will lead to the future design of novel coating surfaces, aiming to produce optimal coating surfaces for biomedical and environmental engineering applications.

## Supplementary Materials

The following supporting information can be downloaded at: https://www.mdpi.com/article/10.3390/molecules27217394/s1, Figure S1. Three surface areas/orientations that are defined in BSA protein: A (color: red), B (color: yellow), and C (color: blue). The structure of the BSA protein is based on X-ray crystallography (PDB id code: 3V03); Figure S2. Initial constructed models by which BSA is placed on poly(SPE) surfaces at three different orientations, at low-density (left) and at high-density (right) surfaces: models LA, LB, and LC (left, from top to bottom), and models HA, HB, and HC (right, from top to bottom); Figure S3. The root-mean-square-deviations (RMSDs) for models LA, LB, and LC at low-density surface (top, from left to right) and for models HA, HB, and HC at high-density surface (bottom, from left to right). The RMSD values were computed separately for bovine serum albumin (BSA) protein (color: black), for the poly(SPE) grafted surface (color: green), and for the combined BSA–poly(SPE) surface model (color: red); Figure S4. The averaged percentage distribution of the number of inter-chain electrostatic interactions within the poly(SPE) zwitterionic side-groups, in absence and in presence of BSA, at low- and high-density surfaces; Figure S5. Snapshots of the detachment and anchoring of BSA simulated models at high-density surface for the three trajectories/replicas (right). Initial models are described in Figure S2. The height values of the BSA from the surface were measured along the Z-component axis for the Cα atoms of amino acids (left). The values presented in the graphs are taken from the lowest Cα atom of the protein. The green bands represent the averaged positions of the polymer chains; Figure S6: Snapshots of anchoring and penetrating of BSA simulated models at low-density surface for the three trajectories/replicas (right). Initial models are described in Figure S2. The height values of the BSA from the surface were measured along the Z-component axis for the Cα atoms of amino acids (left). The values presented in the graphs are taken from the lowest Cα atom of the protein. The green bands represent the averaged positions of the polymer chains; Figure S7. The averaged percentage distribution of the root-mean-square fluctuations (RMSFs) for the polymer chains for the models in the absence of BSA–model H, and in presence with BSA models HA, HB, and HC at high-density (top), and for the models in the absence of BSA–model L, and in presence with BSA models LA, LB, and LC at low-density (bottom); Figure S8. The averaged percentage distribution of the root-mean-square fluctuations (RMSFs) for the each of the three-polymer side chains R0 (color: purple), R1 (color: light blue), and R2 (color: green) for the models in the absence of BSA–model L, and in presence with BSA models LA, LB, and LC at low-density (left), and for the models in the absence of BSA–model H, and in presence with BSA models HA, HB, and HC at high-density (right); Figure S9. The percentage interactions that were computed for model HC. The percentage electrostatic interactions between positively charged amino acids (color: blue), negatively charged amino acids (color: red) within the BSA and the polymer chains. The percentage hydrogen bond interactions between uncharged amino acids within the BSA and the polymer chains (color: green); Figure S10. The averaged percentage distribution of the number of water molecules per side chain R0, R1, and R2; Figure S11. The percentage electrostatic interactions between positively charged amino acids (color: blue), negatively charged amino acids (color: red) within the BSA and the polymer chains. The percentage hydrogen bond interactions between uncharged amino acids within the BSA and the polymer chains (color: green). The interactions were computed for the models by which shown these interactions; Figure S12. The topology heights of the polymer chains on the surfaces at snapshots of 20 ns and 120 ns of the MD simulations, for the models in the absence of BSA–model H, and in presence with BSA models HA, HB, and HC at high-density (left), and for the models in the absence of BSA–model L, and in presence with BSA models LA, LB, and LC at low-density (right); Figure S13. The topology of the RMSF of the polymer chains on the surfaces at snapshots of 20 ns and 120 ns of the MD simulations, for the models in the absence of BSA–model H, and in presence with BSA models HA, HB, and HC at high-density (left), and for the models in the absence of BSA–model L, and in presence with BSA models LA, LB, and LC at low-density (right); Figure S14. The topology of the water hydration of the polymer chains on the surfaces at snapshots of 20 ns and 120 ns of the MD simulations, for the models in the absence of BSA–model H, and in presence with BSA models HA, HB, and HC at high-density (left), and for the models in the absence of BSA–model L, and in presence with BSA models LA, LB, and LC at low-density (right); Table S1. The residues and the total number of charged and uncharged residue within the BSA that face the poly(SPE) surface in each orientation.
