# Supplementary material for: Distinct Antifouling Mechanisms on Different Chain Densities of Zwitterionic Polymers
Source: Molecules. 2022 Oct 31;27(21):7394. doi: 10.3390/molecules27217394 (PMC9654173; doi:10.3390/molecules27217394)
Supplement: Supplementary file 1 [file molecules-27-07394-s001.zip › molecules-1940349-supplementary.pdf]

Supplementary Materials for

"Distinct Antifouling Mechanisms on Different Chain Densities of Zwitterionic Polymers"

Clil Regev [1,2], Zhongyi Jiang [3], Roni Kasher [2,*] and Yifat Miller [1,4,*]

[1]Department of Chemistry, Ben-Gurion University of the Negev, P.O. Box 653, Be'er Sheva 84105, Israel

[3] Department of Desalination and Water Treatment, Zuckerberg Institute for Water Research, Jacob Blaustein Institutes for Desert Research, Ben-Gurion University of the Negev, Midreshet Ben-Gurion, 8499000, Israel

[4] Key Laboratory for Green Chemical Technology of Ministry of Education, School of Chemical Engineering and Technology, Tianjin University, Tianjin, China

[2] Ilse Katz Institute for Nanoscale Science and Technology, Ben-Gurion University of the Negev, Beér-Sheva 84105, Israel

Corresponding authors:

Yifat Miller: ymiller@bgu.ac.il

Roni Kasher: kasher@bgu.ac.il

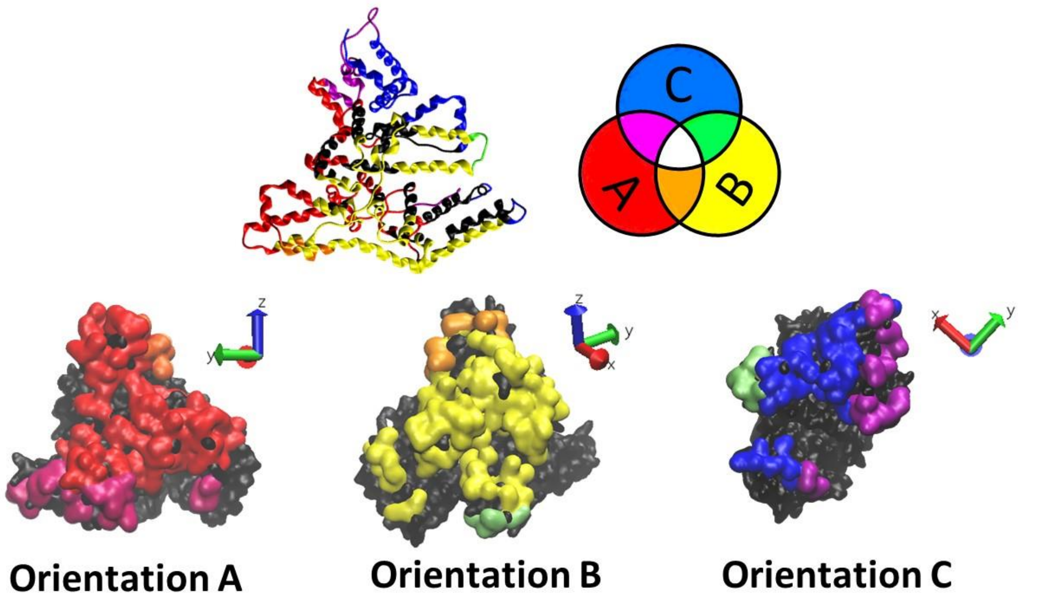

Figure S1. Three surface areas/orientations that are defined in BSA protein: A (color: red), B (color: yellow), and C (color: blue). The structure of the BSA protein is based on X-ray crystallography (PDB id code: 3V03). (Majorek, K.A., Porebski, P.J., Dayal, A., Zimmerman, M.D., Jablonska, K., Stewart, A.J., Chruszcz, M., Minor, W. *Mol Immunol* 52, 174–82 (2012)).

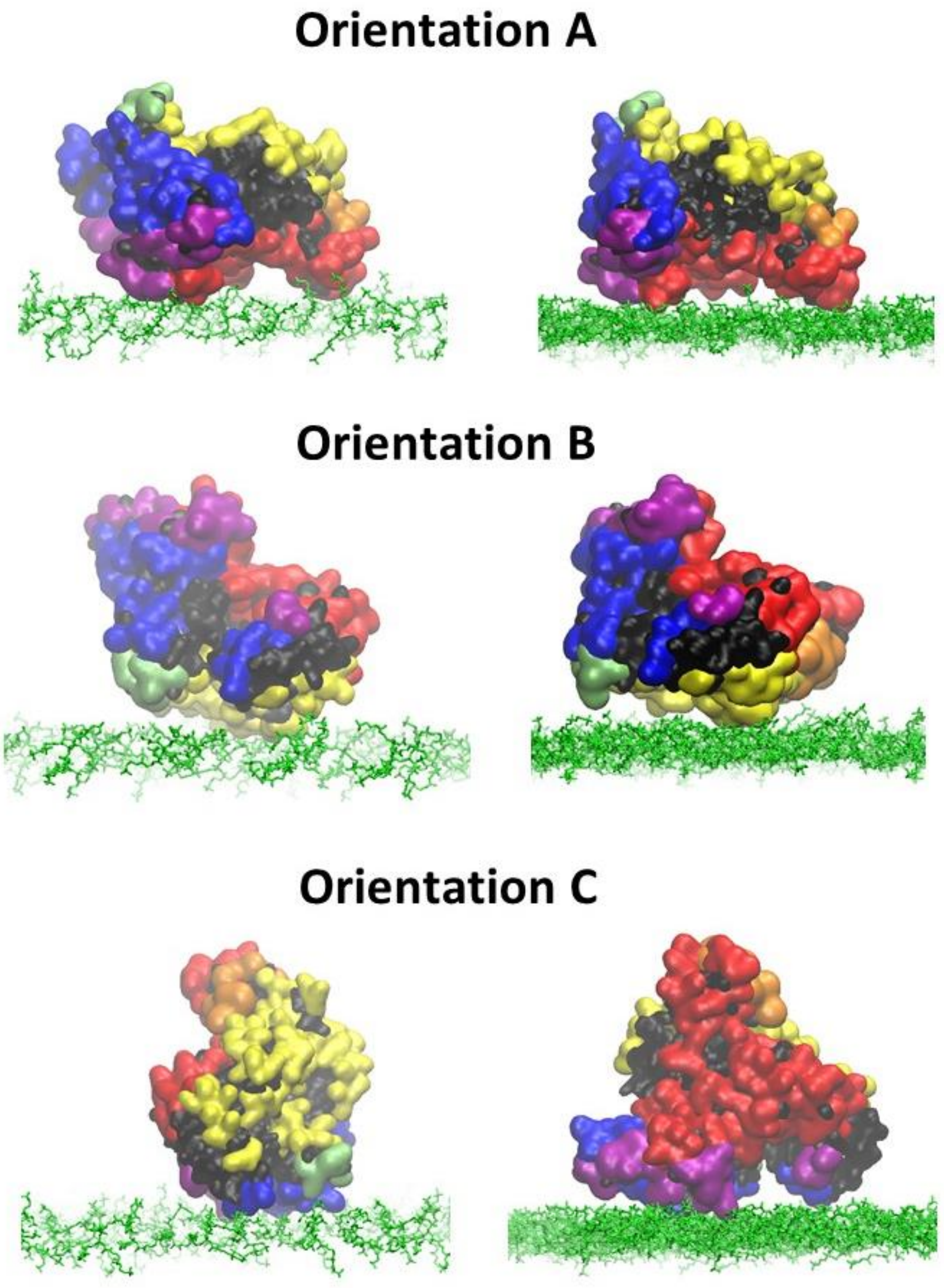

Figure S2. Initial constructed models by which BSA is placed on poly(SPE) surfaces at three different orientations, at low-density (left) and at high-density (right) surfaces: models LA, LB, and LC (left, from top to bottom), and models HA, HB, and HC (right, from top to bottom).

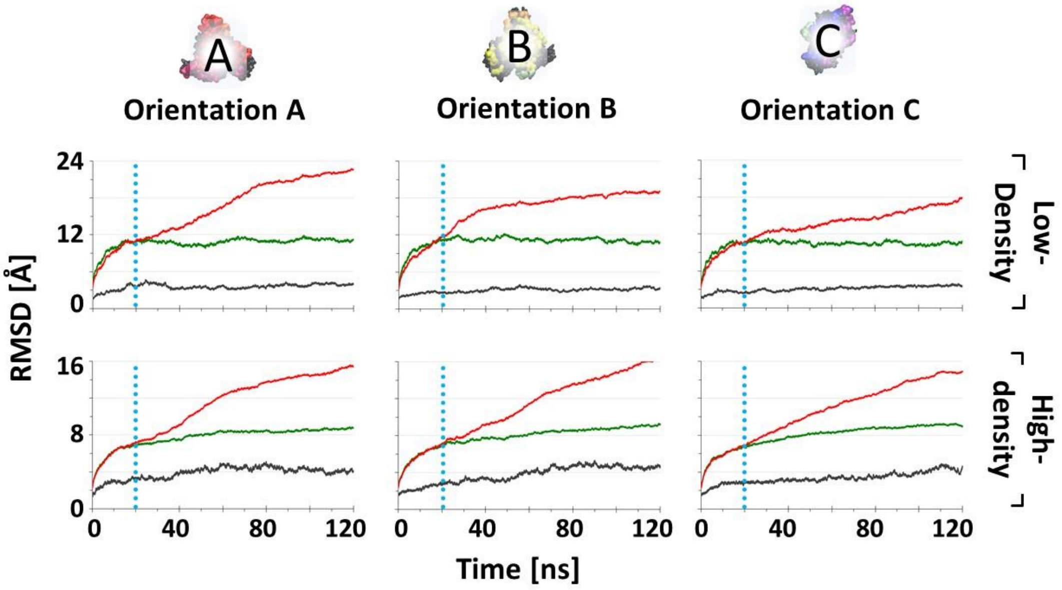

Figure S3. The root-mean-square-deviations (RMSDs) for models LA, LB, and LC at low-density surface (top, from left to right) and for models HA, HB, and HC at high-density surface (bottom, from left to right). The RMSD values were computed separately for bovine serum albumin (BSA) protein (color: black), for the poly(SPE) grafted surface (color: green), and for the combined BSA – poly(SPE) surface model (color: red).

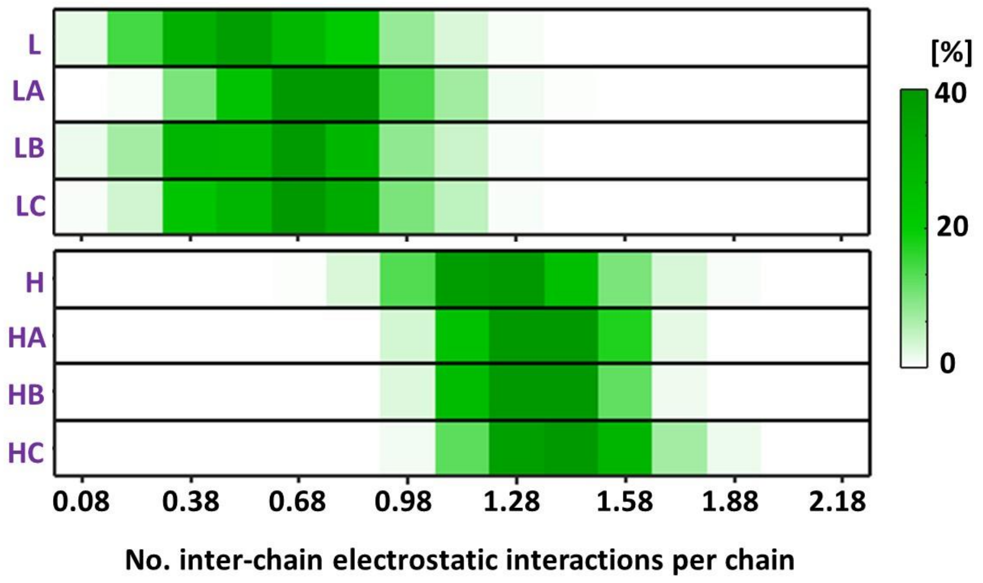

Figure S4. The averaged percentage distribution of the number of inter-chain electrostatic interactions within the poly(SPE) zwitterionic side-groups, in absence and in presence of BSA, at low- and high-density surfaces.

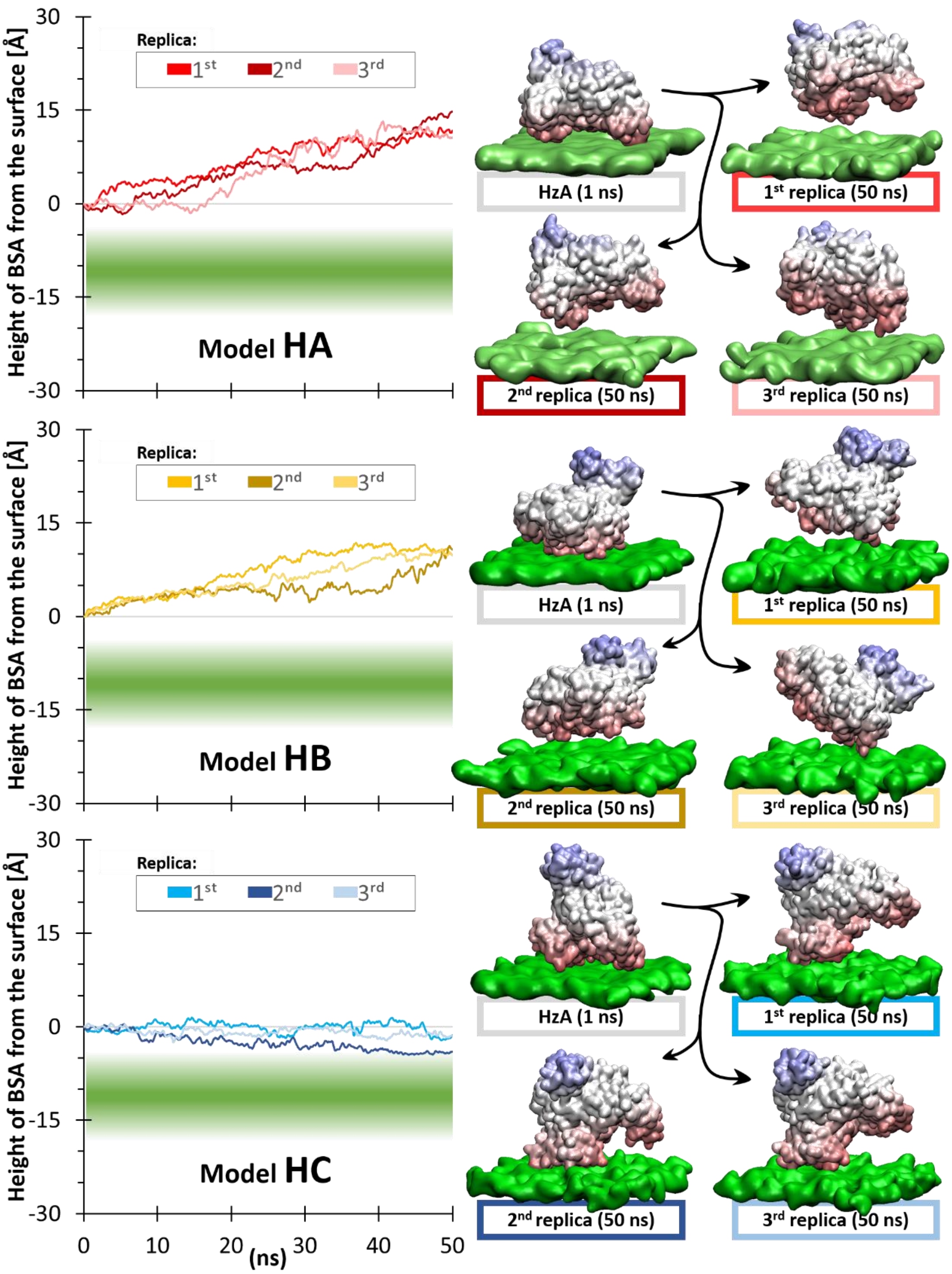

Figure S5. Snapshots of the detachment and anchoring of BSA simulated models at high-density surface for the three trajectories/replicas (right). Initial models are described in Fig. S2. The height values of the BSA from the surface were measured along the Z-component axis for the Cα atoms of amino acids (left). The values presented in the graphs are taken from the lowest Cα atom of the protein. The green bands represent the averaged positions of the polymer chains.

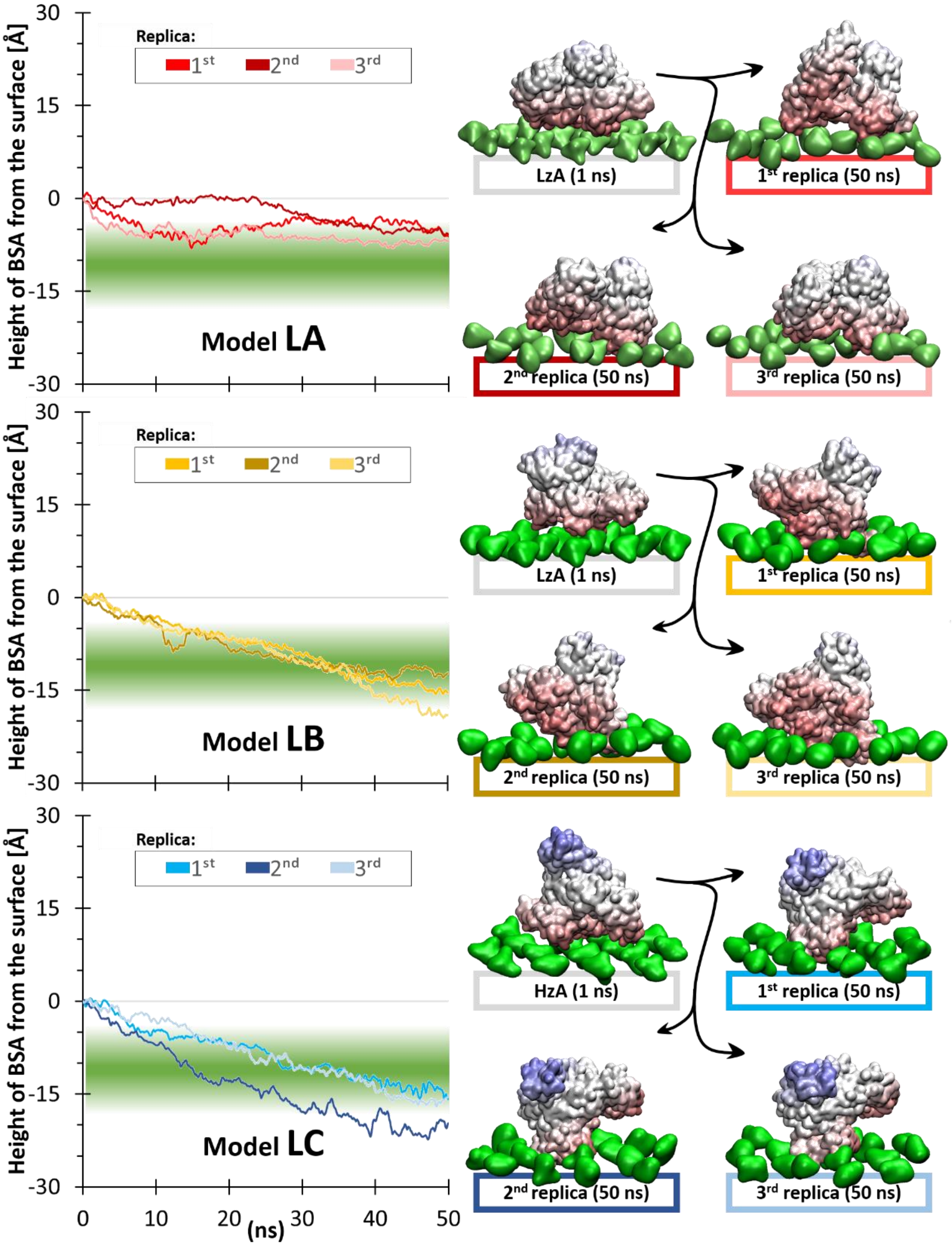

Figure S6: Snapshots of anchoring and penetrating of BSA simulated models at low-density surface for the three trajectories/replicas (right). Initial models are described in Fig. S2. The height values of the BSA from the surface were measured along the Z-component axis for the Cα atoms of amino acids (left). The values presented in the graphs are taken from the lowest Cα atom of the protein. The green bands represent the averaged positions of the polymer chains.

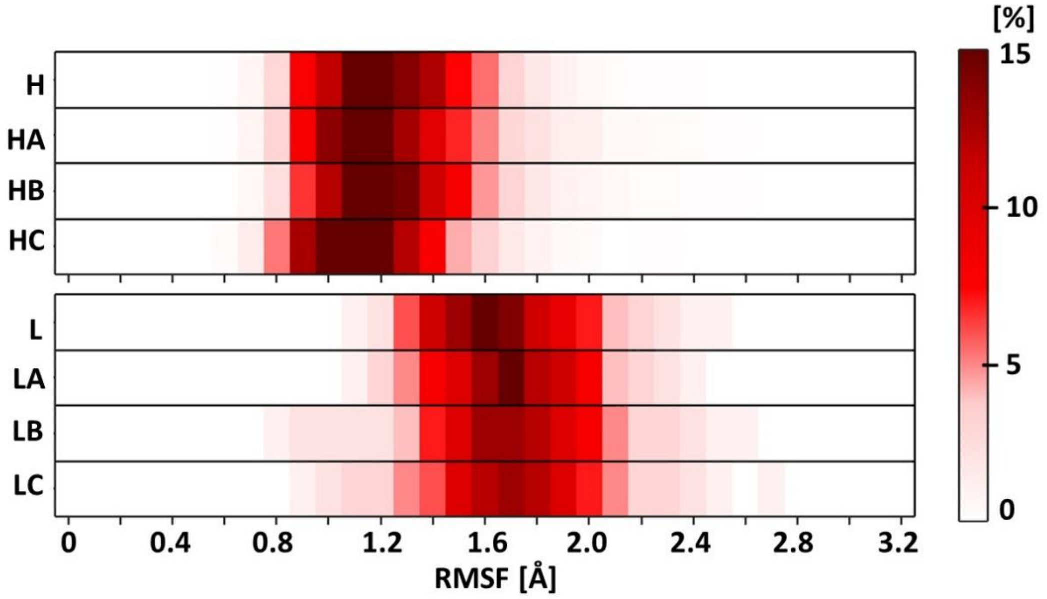

Figure S7. The averaged percentage distribution of the root-mean-square fluctuations (RMSFs) for the polymer chains for the models in the absence of BSA – model H, and in presence with BSA models HA, HB, and HC at high-density (top), and for the models in the absence of BSA – model L, and in presence with BSA models LA, LB, and LC at low-density (bottom).

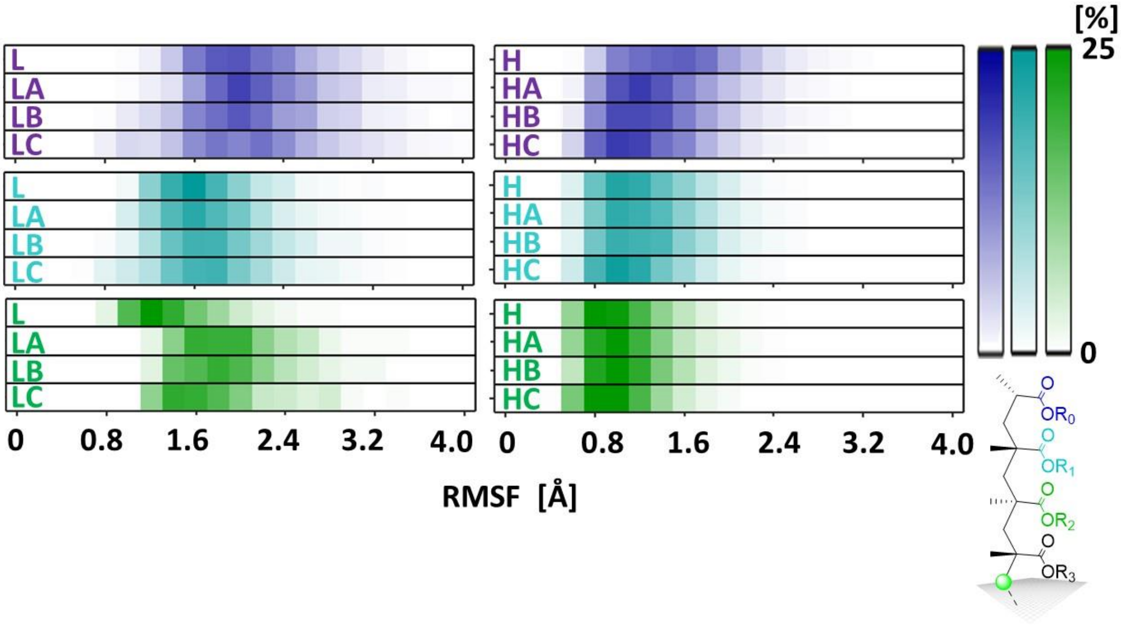

Figure S8. The averaged percentage distribution of the root-mean-square fluctuations (RMSFs) for the each of the three-polymer side chains R_0 (color: purple), R_1 (color: light blue), and R_2 (color: green) for the models in the absence of BSA – model L, and in presence with BSA models LA, LB, and LC at low-density (left), and for the models in the absence of BSA – model H, and in presence with BSA models HA, HB, and HC at high-density (right).

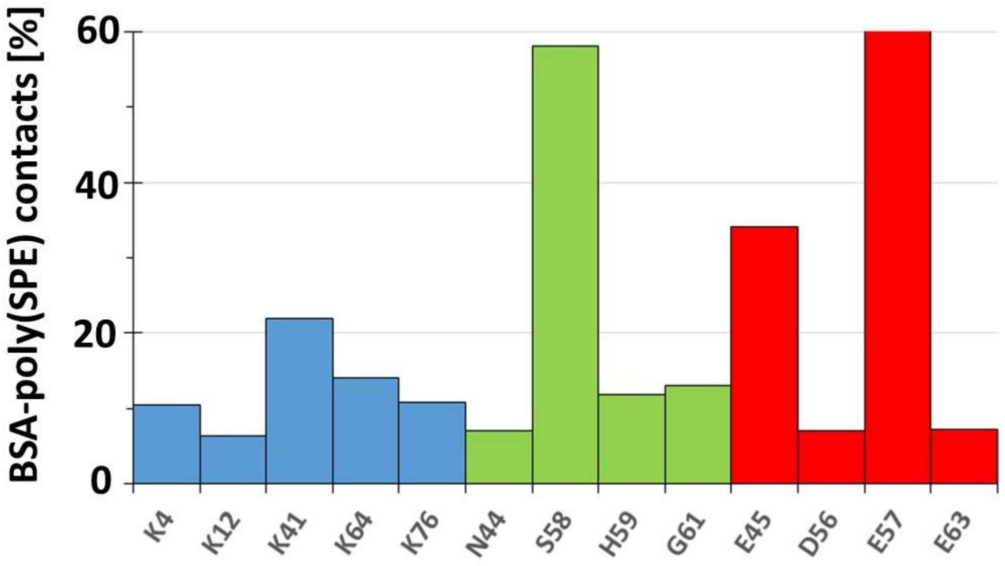

Figure S9. The percentage interactions that were computed for model HC. The percentage electrostatic interactions between positively charged amino acids (color: blue), negatively charged amino acids (color: red) within the BSA and the polymer chains. The percentage hydrogen bond interactions between uncharged amino acids within the BSA and the polymer chains (color: green).

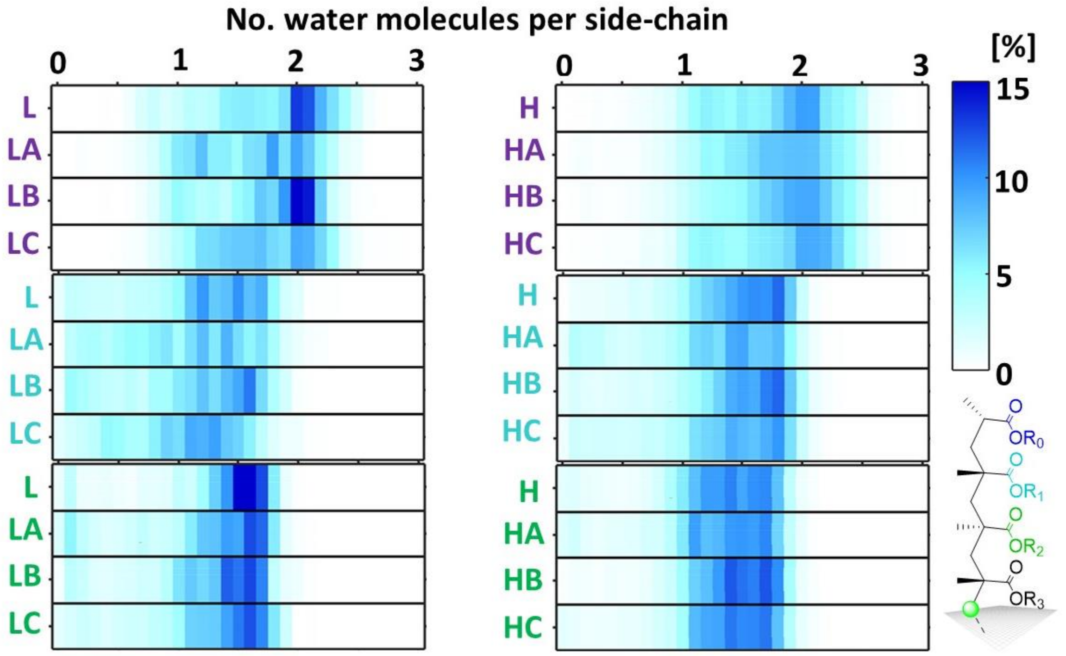

Figure S10. The averaged percentage distribution of the number of water molecules per side chain R_0, R_1, and R_2.

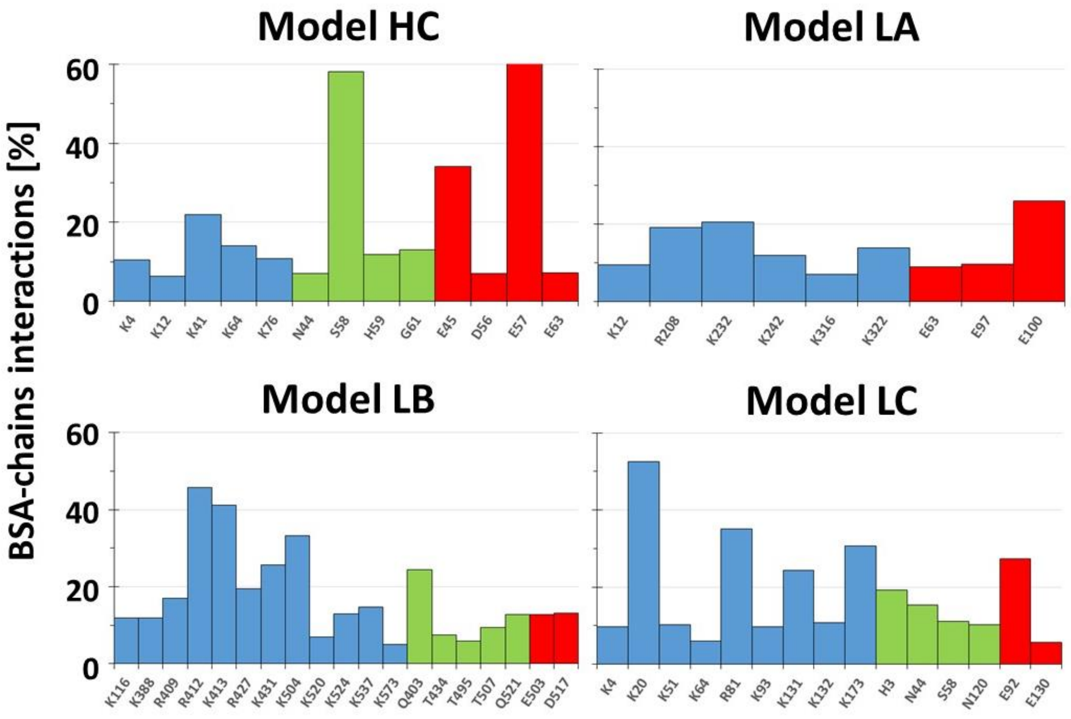

Figure S11. The percentage electrostatic interactions between positively charged amino acids (color: blue), negatively charged amino acids (color: red) within the BSA and the polymer chains. The percentage hydrogen bond interactions between uncharged amino acids within the BSA and the polymer chains (color: green). The interactions were computed for the models by which shown these interactions.

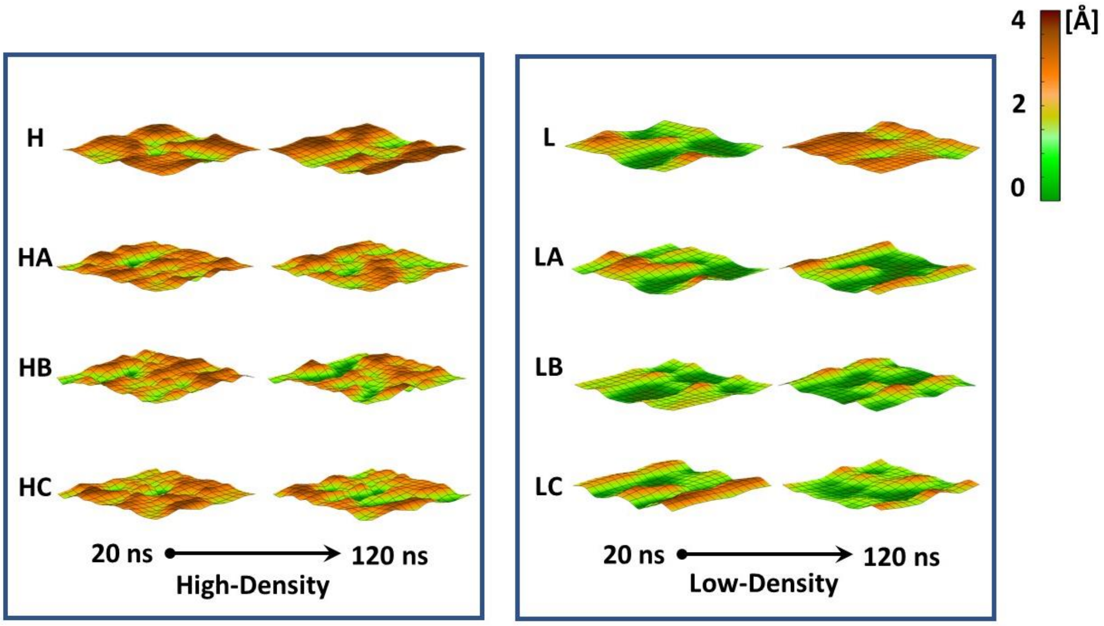

Figure S12. The topology heights of the polymer chains on the surfaces at snapshots of 20 ns and 120 ns of the MD simulations, for the models in the absence of BSA – model H, and in presence with BSA models HA, HB, and HC at high-density (left), and for the models in the absence of BSA – model L, and in presence with BSA models LA, LB, and LC at low-density (right).

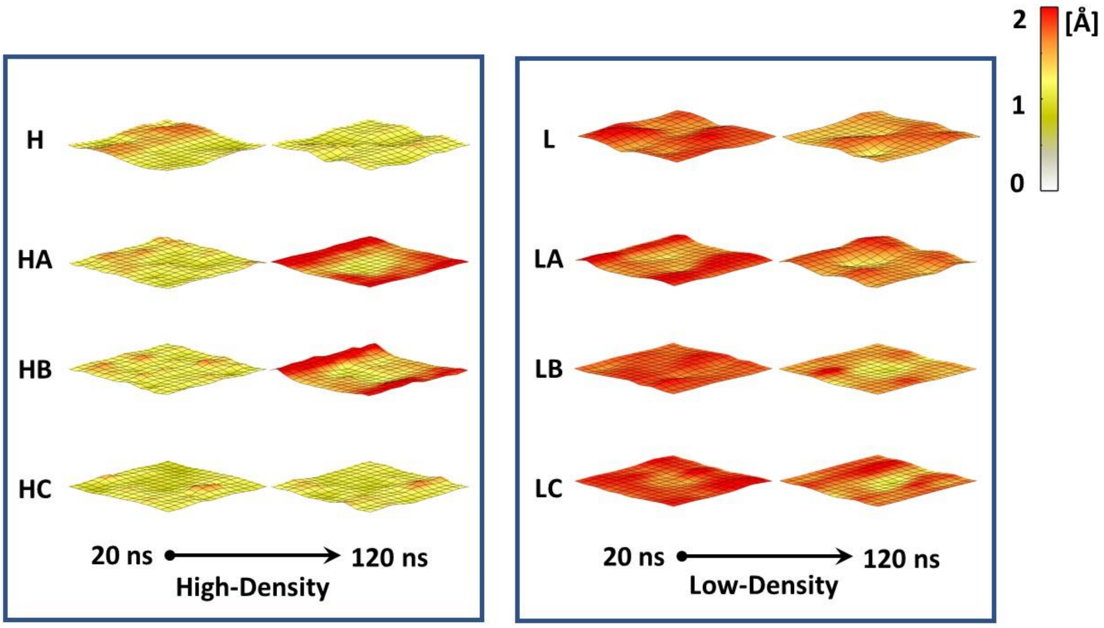

Figure S13. The topology of the RMSF of the polymer chains on the surfaces at snapshots of 20 ns and 120 ns of the MD simulations, for the models in the absence of BSA – model H, and in presence with BSA models HA, HB, and HC at high-density (left), and for the models in the absence of BSA – model L, and in presence with BSA models LA, LB, and LC at low-density (right).

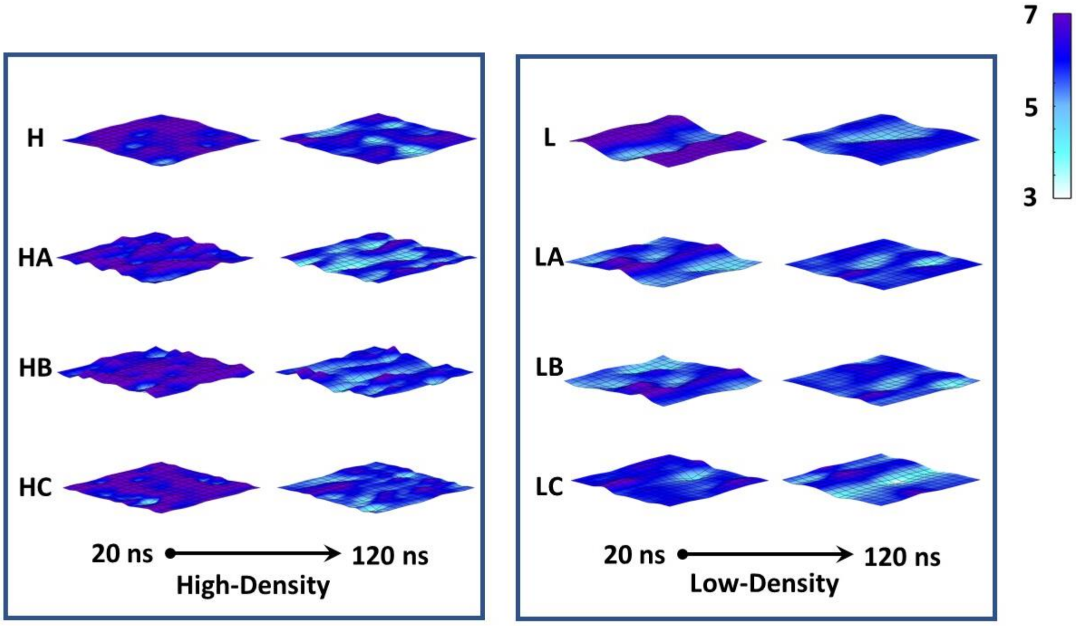

Figure S14. The topology of the water hydration of the polymer chains on the surfaces at snapshots of 20 ns and 120 ns of the MD simulations, for the models in the absence of BSA – model H, and in presence with BSA models HA, HB, and HC at high-density (left), and for the models in the absence of BSA – model L, and in presence with BSA models LA, LB, and LC at low-density (right).

Table S1. The residues and the total number of charged and uncharged residue within the BSA that face the poly(SPE) surface in each orientation.

**Orientation**	**Residues within BSA facing the poly(SPE) surface**	**Number of positively charged residues**	**Number of uncharged H-Donor residues**	**Number of negatively charged residues**
**A**	56 -111<br>199-213<br>240-251<br>307-330<br>347-378<br>411-420<br>458-505<br>532-539	31	51	38
**B**	158-189<br>216-227<br>264-283<br>290-311<br>330-342<br>368-403<br>432-452<br>540-558	23	44	31
**C**	1-92<br>120-139<br>168-174<br>500-511<br>560-565	17	35	29
